# Codon insertion and deletion functions as a somatic diversification mechanism in human antibody repertoires

**DOI:** 10.1186/1745-6150-1-24

**Published:** 2006-08-30

**Authors:** Donald C Reason, Jianhui Zhou

**Affiliations:** 1 Children's Hospital Oakland Research Institute, Oakland, CA, 94609, USA

## Abstract

It has been suggested that codon insertion and/or deletion may represent a mechanism that, along with hypermutation, contributes to the affinity maturation of antibodies. We used repertoire cloning to examine human antibodies directed against 3 carbohydrate antigens and 1 protein antigen for the presence of such modifications. We find that both the insertion and deletion of codons occur frequently in antigen-specific responses following vaccination. Codon insertions and deletions were observed most often in the complementarity determining regions, and less frequently in the framework regions, of VH, Vκ, and Vλ gene segments, and involved motifs known to be preferred targets of somatic hypermutation. Clonal lineage analysis shows that these events occur through out the course of the somatic maturation of individual antibody clones. We also determined that these alterations of paratope structure have varying effects on the relative affinity of the binding site for its cognate antigen.

**Reviewers:**

This article was reviewed by Mark Shlomchik, Deborah Dunn-Walters (nominated by Dr. Andrew Macpherson), and Rachel M. Gerstein.

**Open peer review:**

Reviewed by Mark Shlomchik, Deborah Dunn-Walters (nominated by Dr. Andrew Macpherson), and Rachel M. Gerstein. For the full reviews, please go to the Reviewers' comments section.

## Background

The naïve antibody repertoire arises from the combinational joining of various immunoglobulin gene segments during the antigen-independent maturation of B cells [1]. In the germinal centers (GC) of peripheral lymphoid organs, recently activated B cells encounter accessory cells, T cells, and antigen, and in this environment begin the process of somatic hypermutation (SHM) and class switch recombination (CSR). [2]. SHM introduces non-random point mutations into the variable (V) regions of both the heavy (H) and light (L) gene segments. The mechanism of SHM is incompletely understood, but is known to be mediated by the enzyme activation-induced cytidine deaminase (AID)[3], to target particular sequence motifs with the V gene segments. [2], and is believed to involve double-stranded breaks in the target DNA at the sites modified [4-6]. SHM serves to generate derivative B cell clones with increased, decreased, or unchanged affinity for the stimulating antigen. Clones with increased affinity are presumably preferentially expanded, and give rise to plasma cells secreting serum antibody. These post-rearrangement modifications may also further expand the primary paratopic repertoire available to the host.

In addition to base-pair substitutions, there are several reports of antibodies in which germline V-gene codons have been deleted from the coding region as well as reports in which extra, non-templated codons have been inserted into the coding region of the VH and/or VL gene segments. Such insertions and/or deletions (I/Ds) have been shown to occur in several human B cell malignancies [7-9], in germinal center B cells [10,11], in human hybridomas [12], and in peripheral blood B cells [13]. The co-occurrence of these modifications with base-pair substitutions that appear to have arisen from SHM suggest that I/Ds may be a normal consequence of the somatic maturation of antibody responses. The extent to which I/Ds contribute to antigen-specific responses in humans has not been addressed. In this study we used repertoire cloning to examine human antibodies directed against both carbohydrate and protein antigens for the presence of I/Ds, and find that both events occur frequently in antigen-specific responses following vaccination.

## Results

### Insertions and deletions occur frequently in human antibodies of diverse specificity

We have used repertoire cloning to examine, at the molecular level, the antigen-specific human antibody repertoires directed towards the capsular polysaccharides (PPS) of *Streptococcus pneumoniae *serotype 6B, 14, and 23F [14,15], and the protective antigen (protein) of *Bacillus anthracis *(PA) that arise following vaccination. In the course of this comparative analysis, several antibody Fab fragments were isolated in which the VH or VL regions had been modified through the insertion or deletion of codons as compared to their germline sequence of origin. These modifications (I/Ds) were observed in Fabs specific for PPS 6B (3 of 7 donors), PPS 23F (3 of 6 donors), PPS 14 (2 of 3 donors), and PA (single donor) (Table 1). Taken together, 12 of the 124 independent H and L rearrangements analyzed from these donors (9.7%) contained I/D events. Insertions were 1 or 2 residues in length; deletions varied from 1 to 6 residues in length (Table 2). I/D modifications were found in Vκ, Vλ, and VH regions of the Fabs. Deletions were only noted in the complementarity determining regions (CDRs) while insertions occurred in both the CDRs and frameworks (FR) 1 and 2. The identification of I/D events in the VH CDR3 region is problematic, as this loop often lacks a direct germline element for comparison. The isolation of 10 clonally-related VH 3–23 encoded PPS 6B-specific Fabs from donor 23 has allowed us to determine that deletions occur in this CDR as well. The presence of shared mutations and highly homologous CDR3s indicate a common origin for all of the donor 023 VH3-23 V-gene segments. One clone (023.16B11, Table 3), however, has deleted 4 codons immediately adjacent to the JH5 region.

**Table 1:** Frequency of codon insertions and/or deletions in antigen-specific antibody Fabs isolated from vaccinated donors

Specificity	Donor	Vaccine^a^	#Fabs^b^	Insertions^c^	Deletions^c^
PPS 23F	002	conjugate	3	0	0
	008	conjugate	10	1	0
	014	conjugate	3	0	0
	018	PPS	3	1	0
	023	PPS	3	0	0
	025	PPS	6	0	0
	027	PPS	4	1	0
PPS 6B	001	conjugate	1	0	0
	002	conjugate	5	0	0
	003	conjugate	11	9	0
	010	conjugate	11	0	1
	011	conjugate	20	0	0
	023	PPS	9	1	1
PPS 14	011	conjugate	4	1	1
	012	conjugate	23	0	0
	023	PPS	9	0	4
PA	001	AVA	32	0	1

^a^Vaccine; PPS (23-valent pneumococcal polysaccharide vaccine), conjugate (pneumococcal polysaccharides conjugate to CRM_197_) or AVA (anthrax vaccine). ^b^Number of antigen-specific Fabs isolated from this donor. ^c^Number of Fabs isolated with codon insertions and/or deletions.

**Table 2:** Antigen specificity, V-gene usage, and location of codon insertions and/or deletions in individual antigen-specific Fabs

					Number of Codons	
Specificity^a^	Donors^b^	Clone	Isotype	V gene^c^	Ins/Del	Location^d^
PA	1/1	001.46F1	κ/G1	A27	1 del	CDR1
						
PPS 6B	3/6	003.6F2	κ/G2	VH3-15	2 ins	CDR2
		023.16B11	λ /A2	VH3-23	4 del	CDR3
		010.3H11	κ/A2	B3	6 del	CDR1
		023.20H2	λ/A2	V1-4	1 ins	FR2
						
PPS 14	2/3	023.14H10	λ/G2	VH1-46	1 del	CDR2
		023.9E5	λ/G2	VH1-46	2 del	CDR2
		011.11A11	λ/G2	VH3-7	1 ins	FR1
		011.5G1	κ/A2	VH3-23	1 del	CDR2
						
PPS 23F	3/7	018.6G5	κ/G2	VH3-7	2 ins	FRI
		027.064	κ/G2	VH3-23	1 ins	CDR2
		008.4B7	κ/G2	VH4-4	2 ins	CDR1

^a^Antigen binding specificity of human antibody Fabs. ^b^Number of donors from which Fabs containing insertions or deletions were isolated over the number of donors analyzed. ^c^Germline gene modified in the Fab. Kappa L chain nomenclature as described in [20], lambda L chain nomenclature as described in [21], H chain nomenclature as described in [23]. ^d^Location of the modification; CDR and frameworks regions are as described in [24].

To verify that the I/Ds reported herein were physiologic events and did not arise during the PCR procedures used to produce antibody clones, antibody V regions containing I/Ds were re-isolated from residual cDNA used to make the original libraries. For 2 heavy chains containing deletions (023.9E5 and 023.14H10) and for 1 heavy chain containing an insertion (008.4B7) sufficient material remained following library construction to serve as template for an additional PCR. Primers were designed to hybridize with the unique CDR3 regions of these 3 clones, and used paired with the optimal upstream primer to re-PCR the remaining cDNA. Analysis was limited to H chains in order to take advantage of the unique CDR3 sequences. In all three cases, VH regions identical, both in terms of I/Ds and point mutations, to those reported in Table 3 and 4 were re-isolated from the appropriate cDNA, thereby rendering PCR error unlikely as the source of I/Ds in these clones.

### I/Ds occur at RGYW/WRCY motifs

Single base-pair substitutions that occur within the V regions of immunoglobulin genes during the course of SHM tend to occur at "hotspots" defined by RGYW/WRCY sequence motifs [26]. I/Ds identified in randomly selected immunoglobulin chains have also been associated with these same motifs [27], suggesting that the mechanism that generates I/Ds may utilize the same enzymatic mechanism as the more common point mutations. In this study, the location of I/Ds was highly correlated with RGYW/WRCY motifs (Table 3, 4). All insertions were found to be located within local sequences that satisfied this motif, regardless of the specificity of the Fab or the isotype of the chain modified. However, such sequence motifs occur frequently within the CDRs. It is of note, therefore, that the 3 insertions we identified within the framework regions (clones 011.11A11, 023.20H2, and 018.6G5) also occur within RGYW motifs. Four of the six deletions we identified in this study were also located within RGYW motifs. The analysis of deletions is compromised, however, since it is not possible to know the sequence of the deleted region. In clone 023.16B11, for example, if codon 100h, CTT, had mutated to GTT prior to the deletion, a RGYW motif (AGTT) would have been created at the site of the deletion.

### Insertions duplicate adjacent codons

In all cases inserted codons duplicate those immediately 5' or 3' to the insertion (Table 4). This is valid for single codon insertions (clones 011.11A11, 023.20H2, 027.064) as well as those with 2 codon insertions (clones 003.6F2, 018.6G5, and 008.4B7). This high degree of homology, especially in the case of double insertions, strongly implies that the inserted codon(s) are templated on those immediately adjacent to the site of insertion. In one case (008.4B7) the homology involves a mutated codon, and the insertion may have occurred following the mutation. However, the AGT to AAT mutation in codon 31 of this Fab would have eliminated the imbedded AGTT motif containing the insertion.

### Deletions may also be templated by adjacent codons

In the 3 instances of multiple codon deletions (clones 023.9E5, 010.3H11, and 023.16B11) the most 3' deleted codon is homologous to the codon preceding the deletion (Table 3). For clone 023.16B11 this homology was generated by mutation of the preceding codon (position 100e, Table 3). This homology has been noted by others [12,27], and suggest a mechanism consistent with that originally proposed by Streisinger [28] in which polymerase slippage facilitates the formation of a loop in the template strand that fails to be copied in subsequent rounds of replication. As stated above, however, the analysis of deletions is compromised by the necessity of assuming germline sequence in the deleted region.

### I/Ds occur over the course of SHM

**Figure 1:**
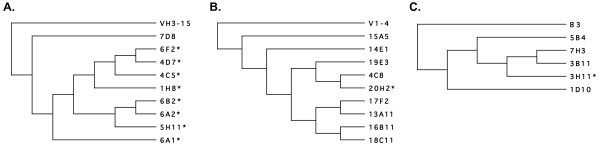
Clonal lineage analysis of selected Fab families showing implied relationships based on shared mutations. **A**) Clustal guide tree generated from an alignment of all clonally related VH3-15 H chains isolated from donor 003 PPS 6B-specific Fabs. Fabs marked with an asterisk (*) all have a 2 codon insertion in the VH CDR2 region. **B**) Clustal guide tree generated from an alignment of all clonally related V1-4 lambda L chains isolated from donor 023 PPS 6B-specific Fabs. Fab marked with an asterisk (*) has a 1 codon insertion in the L chain FR2 region. **C**) Clustal guide tree generated from an alignment of all clonally related B3 kappa L chains isolated from donor 010 PPS 6B-specific Fabs. Fab marked with an asterisk (*) has a 6 codon deletion in the L chain CDR1 region.

In 4 donors, multiple, clonally-related but sequence-unique VH or VL chains were isolated that allowed the analysis of I/D events in the context of ongoing SHM. Three PPS 23F-specific Fabs were isolated from donor 018 that utilized VH3-7 H chains paired with A23-encoded L chains. The VH regions of these Fabs were identical in CDR3, shared 10 mutations as compared to the VH3-7 germline sequence, were rearranged to JH4b, and appear to have arisen from the same initial VDJ rearrangement event. Two of these Fabs had 2 codon insertions in the FR1 region of the H chain (represented by clone 018.6G5, Table 4); the remaining Fab lacked insertions in the VH region. The sequence similarity of the 3 chains suggest that SHM had begun in this clone prior to the insertion event, and continued following the event. From donor 003, 9 unique Fabs were isolated that were specific for PPS 6B and utilized clonally related VH3-15 encoded VH regions. Although unique in sequence, all H chains shared the majority of their mutations, were highly homologous in CDR3, and had rearranged with JH5b. All but one of these chains had a 2-codon insertion in CDR2 (represented by clone 003.6F2, Table 4). The clone lacking the insertion (003.7D8) shared 15 of 28 mutations with other members of the family, as well as the CDR3 characteristics described above. Examination of Clustal multiple-alignment guide trees generated for these sequences (Figure 1A) indicates that the insertion event occurred early in the SHM-induced divergence of the clones. A similar analysis of the V1-4 lambda L chain family (9 unique members) of 6B-specific clones isolated from donor 023 indicates the single chain containing the insertion in FR2 arose late in the differentiation of this clone (Figure 1B). Clone 20H2 shares 23 of 26 mutations with other members of the group, and differs from its closest relative lacking the insertion by only 3 mutations. The insertion event therefore appears to have occurred late in the somatic maturational history of this clone. 5 clonally related B3-utilizing PPS 6B specific clones were isolated from donor 010. One of these (010.3H11, Table 3) had deleted 6 codons in the L chain CDR1. All chains shared an identical insertion at the Vκ/Jκ junction as well as several mutations, and were all rearranged to Jκ1 (an additional B3 isolate from this donor shared no mutations and had rearranged to Jκ4 indicating it was the product of a separate rearrangement event). Clustal alignment and analysis (Fig 1C) indicated that in this series, the deletion event occurred midway through the course of SHM, with mutations accumulating both before and after the deletion of the 6 residues in CDR1. Taken together, the analysis of these 4 Fab families indicates that the I/D events were temporally associated with SHM, and did not occur during V/J and V/D/J rearrangements, when double-strand breaks are known to occur [1].

### I/Ds have varying effect on affinity for antigen

**Figure 2:**
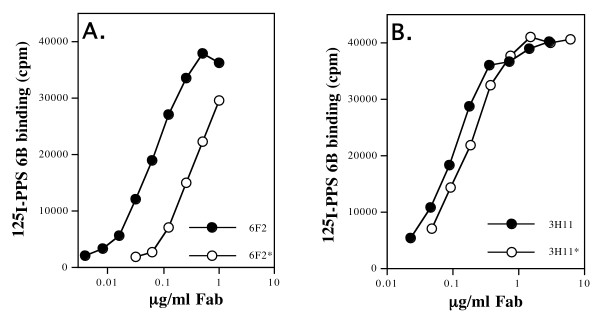
Effect of codon insertion and deletion on antigen binding. Fabs were mutated to either remove inserted codons, or replace deleted codons and the effect on relative affinity for PPS 6B measured. **A**) Relative affinity of Fab 6F2 for PPS 6B with (6F2) and without (6F2*) the 2 codon insertion into H chain CDR2 (see Table 4). **B**) Relative affinity of Fab 3H11 for PPS 6B with a 6 codon deletion in L chain CDR1(3H11; Table 3) compared to that of the Fab with the deleted residues restored to their germline sequence (3H11*).

Insertions and deletions into the CDR regions alter the canonical loop structure of the combining site [29], and have the potential to radically effect antigen binding. To determine the affect such alterations have on relative affinity for the antigen, I/D events were restored to their presumed germline configuration for two selected clones and antigen binding quantitated for the subsequent Fabs. Fab 003.6F2 was engineered to remove the 2 codon insertion in H chain CDR2 and PPS 6B binding measured (figure 2A). The removal of the insertions reduced the relative affinity of the Fab for PPS 6B approximately 5 fold, indicating that the *in vivo *modification had served to generate a more avid combining site. When similar methodologies were used to re-insert the 6 codon deletion in 010.3H11 L chain CDR1 however, little if any effect was seen on the affinity of the Fab for PPS 6B (Figure 2B). As stated above, inferences made from experiment in which deleted residues were restored are compromised, since it is not possible to know the nature of the deleted residues.

**Figure 3:**
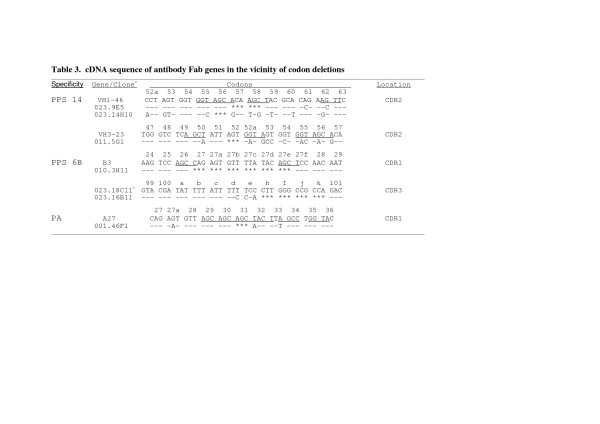
^a^V gene germline sequence aligned with Fab gene sequence in the vicinity of codon deletions.  Dashes indicate   identity with the germline-encoded sequence.   Asterisk (*)  indicate deleted bases.    ^b^The 4 codon deletion in   the CDR3 region of the 023.3H11 heavy chain is shown compared to a clonally related sequence that lacks the   deletion.    Bases  constituting  RGYW/WRCY  motifs  are  underlined.  Codons  are  numbered  as  in[24].    IGMT/HUGO nomenclature  for  the  kappa  light  chain  names  listed  above  is  IGKV4-1  (B3)  and  IGKV3-20   (A27).

**Figure 4:**
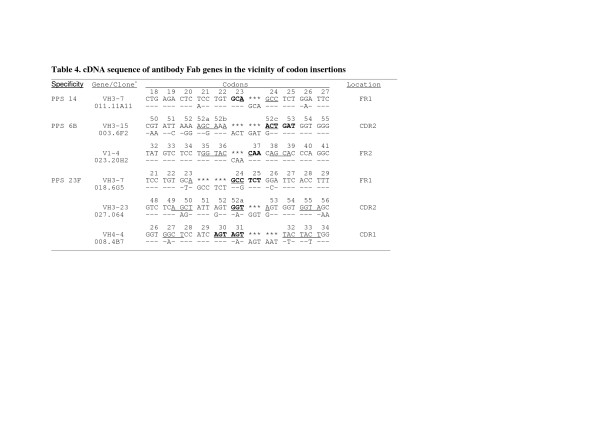
^a^V  gene  germline  sequence  aligned with  Fab  gene  sequence  in  the  vicinity  of  codon  insertions.   Dashes   indicate  identity with  the  germline-encoded  sequence. Asterisks  (*)  indicate  site  of  inserted  bases.   Bases   constituting RGYW/WRCY motifs  are  underlined.   Bases  replicated  in  the  inserted  codons  are  shown  in   bold.  Codons are numbered as  in[24].   IGMT/HUGO nomenclature for  the V1-4 lambda  light chain listed   above is IGLV2-14.

## Discussion

The primary antibody repertoire arises during B cell development through significant structural modifications of the VH and VL gene loci [1]. Through random rearrangement of VH, D, and JH gene segments, and the combinatorial pairing of these rearranged H chains with similarly processed L chains, a "paratope space" is generated that will accommodate a large number of epitopes. There is also evidence that a subset of B cell receptors (BCRs) may be further diversified by the accumulation of point mutations prior to antigenic stimulation. [30]. Secondary BCR diversification occurs in the germinal centers following antigenic stimulation [31-33], and is thought to result primarily from the accumulation of point mutations in the CDRs of both H and L chain V genes. This secondary diversification presumably allows for the selection of antibody clones with increased affinity for the stimulating epitope, and a concomitant increase in antibody efficacy.

Several previous studies have reported immunoglobulin gene sequences in which codons appear to have been inserted into or deleted from the rearranged germline VH and VL genes encoding the mature antibody heterodimer. Although many reports resulted from the examination of B cell malignancies, such modifications have also been reported from non-transformed IgG+ human B cells [27] and human hybridomas [12], and it appears that such modifications may play a physiologically normal role in the somatic maturation of the human antibody response. This argument is strengthened by the observed association of I/Ds both with the CDRs and with motifs within the CDRs known to be preferentially targeted by the enzymatic machinery responsible for somatic hypermutation [34].

Our research utilizes repertoire cloning as a methodology for analyzing antigen-specific antibody response in humans. Repertoire cloning allows us to examine the recombination events and V gene usage that give rise to a protective response for a variety of antigens, to ascertain the degree to which somatic hypermutation modifies the ongoing antibody response to different types of antigens, and to determine the degree to which different members of the population utilize the same mechanisms in generating antibody diversity. These studies offer us the opportunity to examine I/Ds in the context of ongoing antigen-specific response, to compare the response in different donors, and in some cases to locate the I/D event within the SHM history of a single antigen-specific antibody clone. The combinatorial approach we have taken in cloning and screening expression libraries, although highly suitable for repertoire analysis, suffers from the fact that native H and L chain pairing is lost. The frequency with which physiologic Fabs are recreated depends not only on the specificity of B cell enrichment prior to mRNA extraction, but also on the complexity of the antigen-specific antibody repertoire. It is not the case, however, that *de novo *antigen-specific paratopes are created by this methodology. We have shown through direct protein sequencing and idiotypic analysis that the combinatorial cloning technology we employ faithfully reproduces the serum repertoire of polysaccharide-specific antibodies that arise in vaccinated individuals [14,15]. This fidelity is more difficult to demonstrate for protein antigens, however. Protein specific antibody repertoires are diverse, and even a highly enriched protein-specific B cell population would be expected to contain multiple paratopes utilizing several different H and L chains. We cannot rule out that combinatorial pairing in such a complex mixture might give rise to a antigen-specific paratope not present in the *in vivo *population, especially in cases where the majority of the contact residues are located on one chain. Our conclusions pertaining to the single protein specific Fab we present here (PA-specific clone 001.46F1) must therefore be interpreted with this in mind.

We find the somatic modification of antibody genes by the insertion or deletion of codons to be a common occurrence. Of 13 individuals examined in detail for 4 disparate specificities, 8 (62%) utilized I/Ds in the diversification of their repertoires for at least one of the specificities examined. Overall, 9.7% of the independent rearranged V genes analyzed contained I/Ds. All I/Ds we observed were in Ig genes that had undergone some degree of SHM as compared to the germline. It is notable that I/Ds were observed following vaccination both with T-independent (PPS) vaccines, as well as T-dependent vaccines (PPS-protein conjugates and PA). It appears unlikely, therefore, that the T-dependent nature of the immunogen strongly influences I/D events directly. The degree of SHM we observe 7 days following vaccination, however, implies that these are recall responses, and the I/D events we observe (as well as the point mutations) could have arisen earlier during the primary exposure to antigen. The possible T-dependent nature of this primary exposure is unknown. We also confirm here that I/Ds occur within the same RGYW/WRCY motifs known to be hotspots for SHM. Approximately 70% of the bases in the germline VH and VL region reported here lie outside of RGYW/WRCY motifs, making the correlation we observe (100% of insertions and at least 67% of deletions) unlikely to occur by chance alone. Previous reports of I/Ds in randomly selected B cells also noted this association, and support the hypothesis that the enzymatic mechanisms underlying SHM also generate I/Ds. It should be noted, however, that nucleotide sequences satisfying the RGYW/WRCY motif occur commonly in the CDRs of both VH and VL genes, and even if the process is confined to the CDRs by a mechanism unrelated to SHM, association with the RGYW/WRCY motif would be almost unavoidable. However, the fact that the 3 insertions we observed in the framework regions were located within RGYW/WRCY motifs lends significant support to the hypothesis that insertions, deletions, and single base pair mutations are all generated by the same underlying mechanism.

Although incompletely understood, SHM is known to involve AID and low fidelity DNA polymerases. AID is thought to target RGYW/WRCY motifs during transcription, and leads to the deamination of C to U. During repair of this lesion, error-prone polymerases (such as Pol η, Pol ζ, Pol θ, Rev1) would generate mutations which are propagated through further rounds of cell division. Although the proposed method readily accounts for the introduction of point mutations, it is not as obvious how the proposed two-step mechanism could directly generate either insertions or deletions. The fact that insertions duplicate adjacent codons, and that deletion also share probable homology with adjacent codons is consistent with a model of polymerase slippage first proposed by Streisinger [28] as a mechanism for the introduction of frameshift mutations, and this mechanism has been proposed for Ig I/Ds. [13,27]. In this model, unpaired loops form in regions of sequence redundancy during replication, and, depending on which strand the loop forms, an insertion or deletion in the sequence results if the loop is not repaired. This mechanism is not dependent on any particular sequence motif, however, and only requires homology in the vicinity of the I/D site. It cannot in itself explain the predominance of I/Ds in the CDRs and their association with the SHM-preferred motif. There is evidence, however, that both CSR and SHM involve double stranded breaks (DSB) and subsequent rejoining of the coding DNA [4-6,35-37]. Although the role of AID in this mechanism is still undetermined, it has been shown that DSBs occur in the CDRs during SHM, and that these breaks also occur at the SHM-preferred RGYW/WRCY motifs [4]. It has also been shown that DSBs occurring during CSR are staggered [37]. Staggered DSBs in the CDRs during SHM would suggest a possible alternative mechanism for the introduction of both codon insertions (through fill in of overhangs), and codon deletions (through exonuclease trimming of overhangs), and may better account for the restriction of I/Ds to the CDRs than the polymerase slippage model.

The assumption that the I/Ds we describe arise as a consequence of somatic maturation should also be examined in the light of other possible explanations. One is that these are artifactual, that is, they arise either from polymerase error or strand crossover events during the PCR reactions. Our ability to re-isolate the identical V regions containing both the point mutations and the I/D events from residual non-amplified cDNA makes PCR error an extremely unlikely explanation. Another possibility is that an isolated H or L chain containing an apparent I/D event may in actuality represent an un-described allele of the parent V gene. This has been a significant caveat in other studies describing I/Ds. Our ability to place the I/D events in the context of ongoing SHM (i.e. the isolation of clonally-related H and L chains with and without the I/D event) rules this explanation out, at least for those I/D events where several related chains were isolated. Taken together, these two factors strongly support the conclusion that the I/D events we observe represent physiologically relevant events in the clonal maturation of the antibody response.

Alignment and clonal lineage analysis of somatically mutated Ig genes allows us to make several unique observations relevant to the origin and timing of these modifications. In addition to ruling out the involvement of a non-described allele, placing I/Ds within the maturational history of a single rearrangement event allows us to determine that I/Ds can occur during the antigen-driven receptor diversification period of B cell development, and are not restricted to the earlier period of VDJ rearrangement (when DSBs are known to occur). We also show that I/Ds can continue to occur throughout the somatic maturation of the antibody response.

Lastly, we have determined that although I/Ds can dramatically alter the canonical loop structure of the combining site, their effect on antigen binding can be variable, and difficult to predict. The significant deletion of 6 residues from L chain CDR1 in clone 3H11, for example, has little, if any effect on relative binding affinity, while the insertion of 2 residues into H chain CDR2 of clone 6F2 resulted in a significant increase in relative binding affinity. These results are not surprising, however. The contribution of the individual CDRs (and the H and L chains themselves) to the formation of the combining site varies, and an I/D event in a non-contributing CDR (or chain) would be expected to have little effect on affinity for the epitope. And, although our methods of analysis preclude the examination of events that lead to loss of antigen binding, it can be assumed that I/D events also occur that result in a complete loss of affinity as well. A single B cell clone induced to enter SHM by a single antigenic epitope therefore might give rise to a diverse set of descendant clones with minor (through point mutation) and/or major (through codon I/Ds) alterations of the combining sites. A murine model system (the "quasi-monoclonal" mouse) has shown directly that a single VH/VL rearrangement is capable of giving rise to a diverse antibody repertoire through post-rearrangement modifications [38]. I/Ds could therefore be viewed as a mechanism (along with SHM) by which the primary repertoire is diversified to produce BCRs specific for epitopes not covered by the original paratope space generated during combinatorial joining of germ-line gene segments early in B cell development.

The data we present here, when considered with that previously published, provide a compelling argument that the intrachain addition and deletion of codons occurs as a normal part of the somatic maturation of the human antibody response. In addition to increasing antibody efficacy, these significant alteration of paratope structure may also serve to generate a BCR repertoire more diversified than that initially created by VDJ recombination alone. The retention of these modified receptors in the memory pool would significantly expand the range of paratopes available to interact with cognate antigen.

## Materials and methods

The cloning of human antibody repertoires specific for the polysaccharide antigens (PPS) of *Streptococcus pneumoniae *serotypes 23F and 6B have been described in detail previously [14,15]. Fabs specific for PPS serotype 14, and the protective antigen (PA) of *Bacillus anthracis *were isolated using the same approach. In brief, peripheral blood was collected 7 days after vaccination from adult volunteers that had received either the licensed 23-valent polysaccharide vaccine (Pnu-Immune, Wyeth-Lederle) or a 9-valent polysaccharide-protein conjugate vaccine consisting of PPS from serotypes 1, 4, 5, 6B, 9V, 14, 18C, 19F, and 23F conjugated to the mutant diphtheria toxin CRM_197 _(Wyeth-Lederle). For the isolation of PA specific Fabs, blood was collected from a donor 7 days following the sixth dose of the anthrax vaccine AVA (BioPort). Mononuclear cells (MNCs) were isolated from the 7 day post-vaccination blood sample using Ficoll-Hypaque. PPS antigens and PA were biotinylated as previously described [16] and used to "arm" avidin-coated paramagnetic beads (Immunotech Inc., Marseilles, France). These antigen-coated beads were washed, added to 2 × 10^7 ^MNC (pre-absorbed with avidin-coated magnetic beads), and the mixture incubated on ice for 30 min. Antigen binding cells were then isolated with a magnet. The CD19+ percentage of the isolated MNCs ranged from 4 to 23% (average 11%). The number of isolated B cells and the degree of antigen specific B cell enrichment were not determined directly. Positively selected cells were washed twice with cold PBS/0.5%BSA, and used for RNA extraction.

### Construction of Fab expression libraries

The procedures for the construction of Fab libraries have been previously described in detail [14-16]. Briefly, total RNA was prepared from affinity isolated cells (RNAeasy, Qiagen, Valencia, CA) and cDNA prepared using the Thermoscript RT-RCR System (GIBCO BRL, Carlsbad, CA) according to the manufacturers instructions. cDNA was used as template in the polymerase chain reaction (PCR) to generate H chain Fd fragments (VDJ-CH1) and total kappa and lambda L chains for insertion into the expression vector pComb 3H [17] or pARC [14]. Expression libraries generated from a single individual consist, on the average, of about 2 × 10^6 ^clones, of which about >80% produce intact Fabs.

### Identification of antigen-specific Fabs

Individual transformed *E. coli *colonies were selected, mastered onto an LB/carbenicillin agar plate, and grown in 1 ml overnight cultures in deep well 96-well plates under antibiotic selection. Bacteria were pelleted by centrifugation, re-suspended in 140 μl lysis buffer (PBS + protease inhibitor cocktail (Complete, Roche Molecular Biochemicals, Indianapolis, IN)), rapidly frozen and thawed 3 times using liquid nitrogen, and the cellular debris pelleted by centrifugation. Fifty μl of the lysate was transfered to assay plates that had been coated overnight with human light-chain specific antibody (Biosource International, Camarillo, CA) and incubated for 2 hrs at 37°C to facilitate capture of the Fabs. Plates were then washed and 50 μl radio-labeled PPS or PA antigen added to each well. Following incubation at 37°C for 2 hrs, plates were washed, placed on a PhosphorImager detection plate (Molecular Dynamics, Sunnyvale, CA), and the plate was exposed for varying lengths of time. Following exposure, the PhosphorImager plates were scanned, and antigen-binding wells identified. Residual lysate from corresponding clones was re-assayed for binding using a radio-antigen binding assay (for the PPS antigens) or by ELISA (for PA). Positive cultures were identified on the master plates, streaked for isolation, and individual colonies picked and grown overnight. Fab production and antigen-specific binding were then verified in these sub-clones.

### Mutagenesis of selected Fabs

Selected Fabs were mutated either to remove inserted codons or to insert deleted codons. Residues to be modified were selected by comparison to the germline VL or VH gene of origin. Mutations were introduced by primer overlap extension. [18], and verified by sequence analysis.

### Sequencing and sequence analysis

Plasmids containing H and L chain genes were submitted to Davis Sequencing, LLC (Davis, CA) for VH and VL chain sequence determination. Initial sequence analysis utilized the NCBI IgBlast server  to identify candidate germline gene [19]. Following preliminary BLAST alignment, the primary candidate germline gene and all returned near neighbors were manually inspected to ensure both correct germline gene assignment and the correct location of any inserted or deleted codons. Germline genes assignments were made based on the minimum number of mutations required to generate the observed V-gene sequence (maximum parsimony). Subsequent analysis, alignments, translations and clonal lineage analysis were performed using MacVector (Accelrys Inc, Princeton, NJ). To generate the CLUSTALW multiple alignment guide trees, an assumed parent V region was constructed using the known sequence of the relevant V and J germline genes. For heavy chains, a hypothetical CDR3 region was constructed using the most common bases found at each position of the CDR3 in the sequences being analyzed. A distance matrix was generated by pairwise alignment, and these distances used to construct the guide tree that groups and orders the individual sequences. The trees were then "rooted" on the theoretical germline sequence to reflect the origin of the divergent sequences from the original V-(D)-J rearrangement in the naïve B cell. Kappa V region gene nomenclature is as described in [20]. Lambda V region gene nomenclature is as described in [21]. H chain V region gene nomenclature is as described in the IMGT database [22,23]. Complementarity determining regions (CDRs) are as defined in [24].

### Antigen binding and Fab concentration assays

The ability of Fabs to bind antigen was determined by a modified radio-antigen binding assay (for the PPS antigens; [25]) or by elisa (for PA). Fab concentration was determined by a capture ELISA in which goat anti-human Fd (The Binding Site, Birmingham, UK) or goat anti-IgA (Sigma, St. Louis, MO) immobilized on a microtiter plate captures Fab which is then detected by alkaline-phosphatase labeled goat anti-human L chain (Biosource International, Camarillo, CA). This assay is standardized with a purified Fab standard whose concentration was calculated from UV absorbance at 280 nm.

### Genbank accession numbers

All sequences are available from Genbank with the following accession numbers: (clone(H/L) [accession number]): **PPS 6B-specific Fabs: **003.1H8H [Genbank:AY423169], 003.4C5H [Genbank:AY423170], 003.4D7H [Genbank:AY423171], 003.5H11H [Genbank:AY423172], 003.6A1H [Genbank:AY423173], 003.6A2H [Genbank:AY423174], 003.6B2H [Genbank:AY423175], 003.6F2H [Genbank:AY423176], 003.7D8H [Genbank:AY423177], 010.3H11L [Genbank:AY749158], 010.3B11 [Genbank:AY749165], 010.1D10L [Genbank:AY423231], 010.5B4L [Genbank:AY423237], 010.7H3L [Genbank:AY423240], 023.16B11H [Genbank:AY749157] 023.17F2L [Genbank:AY749163], 023.16B11L [Genbank:AY749164], 023.13A11L [Genbank:AY423262], 023.14E1L [Genbank:AY423263], 023.15A5L [Genbank:AY423264], 023.18C11L [Genbank:AY423265], 023.19E3L [Genbank:AY423266], 023.20H2L [Genbank:AY423267], 023.4C8L [Genbank:AY423268]

**PPS 23F-specific Fabs: **027.064H, [Genbank:AF485427], 018.P6G5H, [Genbank:AF485435], 008.4B7H, [Genbank:AF485469], **PPS 14-Specific Fabs**: 023.14H10H [Genbank:AY749159], 023.9E5H [Genbank:AY749160], 011.11A11H [Genbank:AY749161], 011.5G1H [Genbank:AY749162], **PA-Specific Fab**: 001.PA.46F1 [Genbank:AY749156].

## Abbreviations

AID activation-induced cytidine deaminase

AVA anthrax vaccine absorbed (BioThrax)

BCR B cell antigen-specific receptor

BLAST Basic Local Alignment Search Tool

CDR complementarity determining region

CRM197 mutant diphtheria toxin

CSR class switch recombination

DSB double stranded breaks

Fab fragment antigen binding

Fd heavy chain fragment containing the VDJ and the first constant domain

GC germinal center

H heavy

I/Ds insertion and/or deletion

L light

LB Luria-Bertani

MNCs mononuclear cells

PA protective antigen of *Bacillus anthacis*

Paratope combining site of antibody molecule

PBS phosphate buffered saline

PPS pneumococcal polysaccharide

RGYW (A or G) G (C, U, or T) (T, U, or A)

SHM somatic hypermutation

VH variable region of heavy chain

Vκ variable region of kappa light chain

Vλ variable region of lambda light chain

WRCY (T, U, or A) (A or G) C (C, U, or T)

## Competing interests

The author(s) declare that they have no competing interests.

## Authors' contributions

Donald Reason and Jianhui Zhou contributed equally to research planning, experimental procedures, data interpretation, and manuscript preparation. All authors read and approved the final manuscript.

## Reviewers' comments

We would like to sincerely thank the reviewers for their time, their effort, and their helpful suggestions.

### Reviewer's report 1

Mark Shlomchik, MD, PhD, Professor of Laboratory Medicine and Immunobiology, Yale University School of Medicine, New Haven, CT, USA

This paper by Reason and Zhou is an interesting analysis of the sequences obtained from a carefully designed repertoire cloning exercise, using PBL CD19+ cells enriched for specificity for immunizing Ags. Volunteers had received various vaccines 7 days prior to blood collection. An interesting aspect is the focus on carbohydrate-specific epitopes, which are more constrained in repertoire and thus more likely to reproduce authentic VH/VL pairs even though repertoire cloning re-associates these at random in vitro.

Though there are potentially a number of important features of these sequences, the current manuscript focuses on the somewhat unexpected finding that V sequences contain insertions and deletions at a relatively high frequency. Actually, as acknowledged and partly referenced, small insertions and deletions (I/D's) have long been known to accompany somatic hypermutation, though they have generally been noticed in the context of noncoding or else inactivating mutations that would lead to frameshifts. Nonetheless, this work shows that such I/D's are often in frame and can be quite long, and are often compatible with maintaining specificity for immunizing Ag. Particularly compelling are the findings of I/D's in the context of clonal lineages, showing they occur after the onset of SHM and that SHM itself can continue after the I/D's. This in turn firmly links the process to Ag-driven SHM in vivo, a point strengthened by the re-isolation of the same I/D's from the original cDNA pools. In addition, limited but interesting reconstruction of I/D's in vivo indicate that they can be neutral or even improve Ag-binding. Overall, this work helps to establish the physiological relevance of I/D's to the development of the Ag-driven repertoire.

The presentation of the data is clear and the interpretations are reasonable.

The genealogies would be better presented with the mutations on them. My analysis of the primary data from tree 1a indicates that there are potentially many independent parallel mutations. More information is needed on how frequent these were and how they were resolved in making the genealogies. A careful analysis of these may reveal PCR hybrids as well.

I declare that I have no competing interests.

### Response to reviewer

*The reviewer expressed concerns over the methodology used to generate the geneologies as well as their presentation. The guide trees were generated using the CLUSTALW multiple alignment algorithm. An assumed parent V region was constructed using the known sequence of the relevant V and J germline genes. For heavy chains, an assumed CDR3 region was constructed using the most frequent bases found in the sequences being analyzed. All sequences were then compared to each other in a pair-wise fashion to determine their degree of divergence from each other, and their similarities stored in a matrix as a distance measurement that reflects the "evolutionary" distance between the individual pairs. From the distance matrix, a guide (phylogenetic) tree is constructed that groups and orders the individual sequences. The trees are then "rooted" on the theoretical germline sequence to reflect the origin of the divergent sequences from the original V-(D)-J rearrangement in the naïve B cell. A section has been added to the materials and methods to better explain the method by which the alignment trees were generated. The suggestion was also made that a better presentation of the guide trees would be to include the mutations on them. Since these V regions are extensively mutated (some with >40 base changes), it is difficult to include the mutations on the trees in a manner that would be informative. The main purpose of the guide trees is to order the divergence of the individual chains such that an inference can be made as to when in the process the I/D events occurred. The reviewers comments regarding crossover events during PCR point to a significant problem when performing PCR on mixed antibody preparation and were raised by other reviewers as well. This is perhaps more an issue for individual mutations than for the generation of insertions and deletions. Although difficult to rule out entirely, examination of related sequences reveled no obvious example of this occuring. More significantly, our ability to re-isolate sequences identical to those reported from residual material that had not been previously manipulated strongly suggest that, at least for these sequences, PCR cross over was not an issue. For other sequences, it remains a possibility and represents a limitation of the reported data*.

*The reviewer is also correct in noticing that the primary sequence data from which the trees were generated indicates several incidences of independent parallel mutations. It must be remembered that these sequences have been subjected to antigen-driven selection, both physiologically during affinity maturation, and technically during the cloning procedures. Only those mutations that retain antigen binding will be detected. We conclude that those incidences of parallel mutation identify residues that directly contribute to antigen/antibody binding, and are therefore positively selected. We have chosen not to include a discussion of them herein only because they are not directly relevant to the main topic of the manuscript. They have been discussed to some degree in the manuscript in which they were originally reported, and are included in another we are preparing that deals with the somatic maturational process of carbohydrate specific antibodies in general*.

### Reviewer's report 2

Deborah Dunn-Walters, Senior Lecturer, Department of Immunobiology, King's College London School of Medicine, Guy's Campus, London (nominated by Dr. Andrew Macpherson, McMaster University Medical Centre, Hamilton, Ontario, Canada)

The authors have used repertoire cloning to isolate antigen specific human Ig genes after vaccination with polysaccharide (both T dependent and T-independent antigens) and protein vaccines. Their analysis of the Fab repertoires produced shows that insertions and deletions (I/Ds) are a fairly common occurrence during affinity maturation of the Ig gene. The observation of I/Ds has been made previously, but not in the context of *specific *antibody responses against different types of antigens. What is nice about this paper is that the authors have isolated clonally-related sequences so that they can create lineage trees and show that I/Ds can occur *during *the affinity maturation process. This, in conjunction with the observations that a) only mutated Ig genes have I/Ds and b) the somatic hypermutation (SHM) hotspots RGYW and WRCY are usually found in the vicinity of the I/Ds, shows that the I/Ds are likely to be created as part of the overall SHM process.

In the discussion, paragraph 5, concerning the theory that polymerase error might be responsible for I/Ds, the authors briefly mention that fidelity of polymerase might be somehow be altered only near RGYW sequences and then they quickly move on to discuss double strand breaks. The current two step hypothesis for the mechanism of SHM is that the initial targetting of WRCY/RGYW by AID causes a mismatch lesion by deamination of C to U. This is thought to trigger the second phase of hypermutation which involves mismatch repair and DOES involve error prone polymerases such as Pol η, Pol ζ, Pol θ, Rev1. Hence linking hotspots with polymerase error is a plausible theory and the error-prone pols should be mentioned.

The authors also reverted two of the clones to replace the (assumed) deletion or to remove the insertion. In one instance the reversion had no significant effect on affinity, whereas the other had a larger effect. This is, as the authors point out, not surprising – as not all changes during affinity maturation will result in significant differences in function. Nonetheless it is interesting that the reversion causing a large difference in affinity was of a conserved insertion that occurred close to the root of the lineage tree, and presumably early in the affinity maturation process, whereas the reversion that didn't make any difference was of a mutation that had occurred slightly later in the affinity maturation process.

I declare that I have no competing interests.

### Response to reviewer

*The reviewer is correct in stating that the currently accepted model of AID-mediated SHM was given insufficient emphasis in our attempt to postulate a mechanism responsible for the generations of I/Ds, and we have added wording to the discussion section of the manuscript we hope will correct this. We do feel, however, that while the currently accepted two-step mechanism of SHM very plausibly explains point mutations in the V regions of immunoglobulin genes, it is less robust in explaining the generation of multi-base insertions and deletions at these same motifs*.

### Reviewer's report 3

Rachel M. Gerstein, Ph.D., Associate Professor, University of Massachusetts Medical School, Worcester, MA, USA

Somatic hyper-mutation (SHM) is an important mechanism for diversifying antibodies produced during an immune response to infection. This is important for "fine-tuning" responses as they evolve and generating higher affinity B cell clones that can effectively compete for and capture antigen as antigen concentration drops off once antibodies and other effector mechanisms make progress in clearing infectious organisms.

Most mutations made during SHM are nucleotide substitutions. This Ms analyzes the less frequent occurrence of insertions and deletions (I/Ds). I/Ds have been observed previously, and, when the antibody gene is left "in-frame", have the ability to modify antigen binding. The unresolved issue to which this Ms contributes is the extent to which I/Ds contribute to antigen-specific responses, and whether I/Ds can improve antigen biding. Importantly, this study considers mutations in human B cells that are generated in response to the clinically important polysaccharide antigens (PPS) of *Streptococcus pneumoniae *and protective antigen (PA) of *Bacillus anthracis*. Studies of SHM in mouse B cells are much more numerous, and studies of mutations in human B cells that arise during responses to infection or vaccines are still limited.

This paper will be of interest to immunologists, particularly those studying SHM. The antigen-specificity of these antibodies allows the authors to construct relational trees from (probably) clonally-related VH or VL chains, and document that I/Ds, like SHM-generated substitutions, are likely to occur over the course of SHM.

The strength of the approach used is that the authors have an effective system, repertoire cloning, to "capture" and analyze antigen-specific immunoglobulins in an unbiased manner: all expressed H chain and L chain genes are cloned by PCR into expression vectors which are then used to transform *E. coli*. lysates from this library are then screened for both Fab content and then specific antigen binding.

Another strength of the approach and the study is that the authors were able to study the contribution of insertions to relative affinity to antigen by mutating the molecular clones so as to restore germ-line sequence, and then measuring antigen binding in the different clones. In one clone, removal of insertions reduced binding 5-fold. It would be interesting to know the affect of other insertions on other clones, as conclusions from one clone are somewhat limited.

#### A number of criticisms are suggested for consideration

The frequency of I/Ds should be presented in a more informative way. Table 1 reports number of Fabs with I or Ds compared to the # of Fabs sequenced for each donor. It would be useful to also report the frequency (ie # nucleotides changed by I/Ds vs the # sequenced).

Similarly, the authors report that location of the I/Ds are highly correlated with RGYW/WRCY motifs (hotspots for SHM), yet no numeric or statistical comparisons are provided. Important in this type of comparison is the frequency overall of RGYW/WRCY motifs in the V gene.

Pooled B cells were used for RNA isolation and subsequent PCR cloning. There is no way to assure that any given sequence is not a product of *in vitro *strand exchange during PCR (particularly problematic for related V-regions; see Ford *et al. Gene *142:279-283, 1994) and this limitation should be acknowledged.

What is the direct evidence that I/Ds are a product of SHM? Typically, specificity and errors introduced by Taq are accounted for by sequencing germ-line genes from the same donor or sequencing a different gene (even CH1 from Cμ). And what argues against the possibility that some sequences can represent allelic variants in the human population?

### Response to reviewer

Tables 1 and 2*were designed primarily to provide the reader with indication of the number of donors we had processed, the depth to which each had been explored, and the general distribution of the I/D events. We agree with the reviewer that it is not easy to ascertain the overall frequency of I/D events from the tables presented. We have therefore re-calculated the overall frequency of I/Ds in this study by determining the total number of independent heavy and light chain rearrangements analyzed for all donors. This eliminates clonal derivatives which would bias the denominator. We find the number of donors in which I/D events to be 8/13 (62%). 12 of the 124 independent H and L rearrangements analyzed from these donors (9.7%) contained I/D events. A sentence stating these frequencies has been added to both the results and discussion section of the manuscript*.

*The caveats of *in vitro *strand exchange during PCR and other PCR-related errors is well taken and was raised by other reviewers as well. To verify that the sequences we report did not arise from PCR related artifacts we decided to re-isolate the antibody V regions containing I/Ds from the residual cDNA used to make the original libraries. This is the most direct verification of the validity of our reported sequences. Analysis was limited to H chains in order to take advantage of the unique CDR3 sequences. For 2 heavy chains containing deletions (023.9E5 and 023.14H10) and for 1 heavy chain containing an insertion (008.4B7) sufficient material remained following library construction to serve as template for an additional PCR. In all three cases, VH regions identical, both in terms of I/Ds and point mutations, to those reported in *Table 3 and 4*were re-isolated from the appropriate cDNA, thereby rendering PCR error unlikely as the source of I/Ds in these clones. We cannot exclude the possibility that other clones may contain PCR-related artifacts. We do, however, believe that the re-isolation of these sequences from non-manipulated material strongly support the conclusion that the I/D events we observe represent physiologically relevant events in the clonal maturation of the antibody response. A paragraph has been added to both the results and to the discussion section of the manuscript to report these new findings*.

*The reviewer also suggest the possibility that some sequences may represent allelic variants of the germline genes in the database. This has been a of particular concern in other studies describing I/Ds since usually only single sequences are reported. In 4 donors, multiple, clonally-related but sequence-unique VH or VL chains were isolated that allowed the analysis of I/D events in the context of ongoing SHM. Our ability to place the I/D events in the context of ongoing SHM (i.e. the isolation of clonally-related H and L chains with and without the I/D event*, figure 1*and 018.6G5) rules this explanation out, at least for these 4 I/D events where several related chains were isolated. We cannot rule this explanation out for sequences that were isolated without clonal relatives lacking I/D events, and this is a limitation of the study*.
